# Supplementary material for: Causal interplay between lactose intolerance and gut microbiota: a combined bidirectional Mendelian randomization and in vivo validation study
Source: Front Nutr. 2026 Jun 1;13:1803337. doi: 10.3389/fnut.2026.1803337 (PMC13265576; doi:10.3389/fnut.2026.1803337)
Supplement: Supplementary file 1 [file Data_Sheet_1.zip › supplementary materials/Forward/forest plot/ebi-a-GCST90027847.finngen_R12_E4_LACTONAS.pdf]

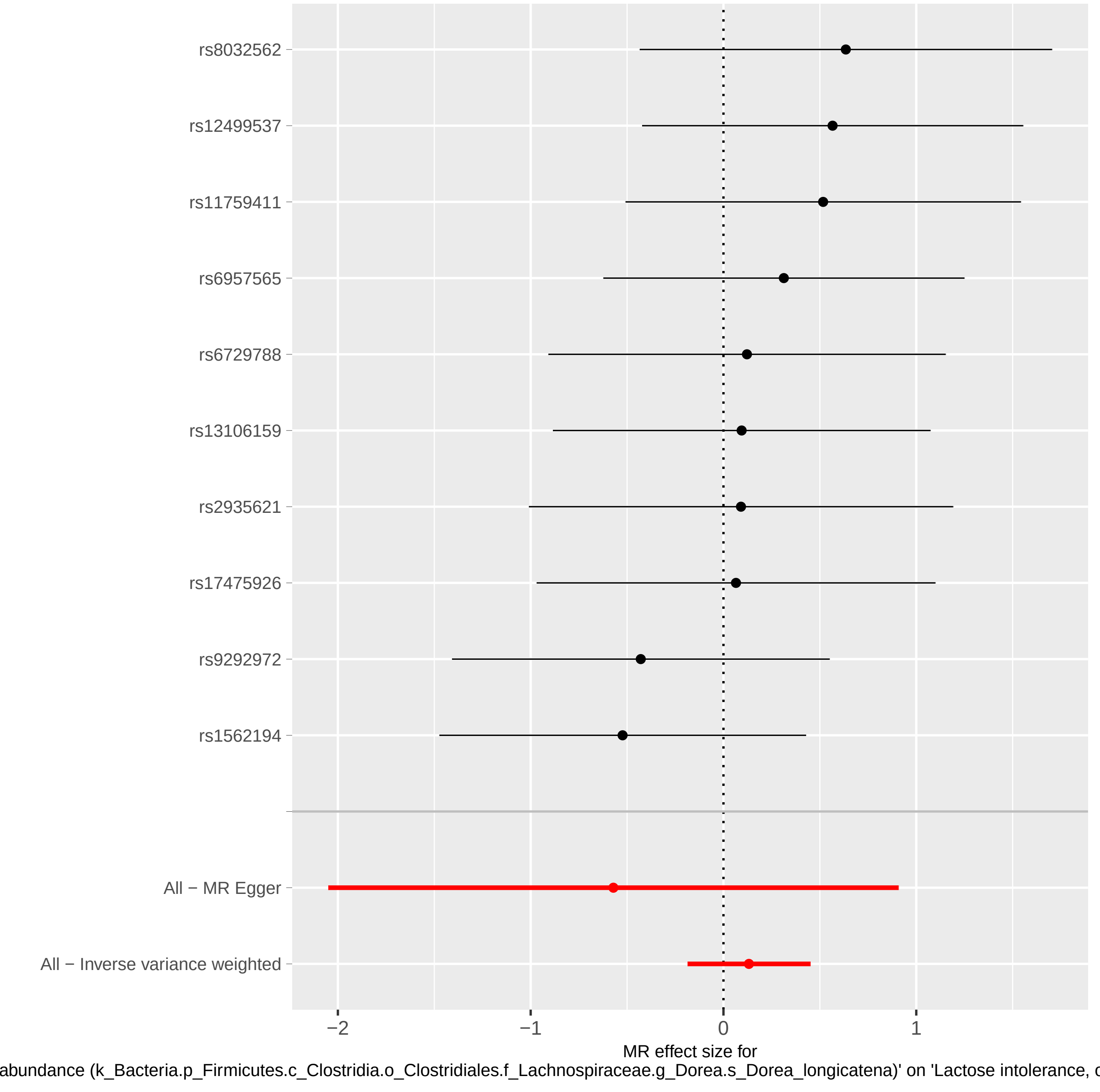
rs8032562
rs12499537
rs11759411
rs6957565
rs6729788
rs13106159
rs2935621
rs17475926
rs9292972
rs1562194
All − MR Egger
All − Inverse variance weighted
−2
−1
0
1
MR effect size for
abundance (k_Bacteria.p_Firmicutes.c_Clostridia.o_Clostridiales.f_Lachnospiraceae.g_Dorea.s_Dorea_longicatena)' on 'Lactose intolerance, c
